# Active Surveillance for Low- and Favourable Intermediate-Risk Prostate Cancer: A Single-Centre Cohort Study on Discontinuation Rates and Quality-of-Life Outcomes

**DOI:** 10.3390/jpm16080420

**Published:** 2026-08-07

**Authors:** Ioannis Mykoniatis, Athanasios Papatzelos, Damianos Damon Dejan Nikolaou Nikolovski, Asterios Symeonidis, Christos Roidos, Chrysovalantis Toutziaris, Ioannis Vakalopoulos, Petros Sountoulides

**Affiliations:** 1 First Department of Urology, “G.Gennimatas” General Hospital, Faculty of Medicine, School of Health Sciences, Aristotle University of Thessaloniki, 54635 Thessaloniki, Greece; g_mikoniatis@hotmail.com (I.M.); symeaste@gmail.com (A.S.); drchriroid22@gmail.com (C.R.); toutziaris@yahoo.co.uk (C.T.); vakalj@otenet.gr (I.V.); 2 Second Department of General Surgery, “G.Gennimatas” General Hospital, Faculty of Medicine, School of Health Sciences, Aristotle University of Thessaloniki, 54635 Thessaloniki, Greece; athpapatzelos@gmail.com; 3 Department of Cardiothoracic Surgery, General University Hospital of Ahepa, Faculty of Medicine, School of Health Sciences, Aristotle University of Thessaloniki, 54636 Thessaloniki, Greece; damianosnikolaou@icloud.com

**Keywords:** active surveillance, prostate cancer, discontinuation, treatment-free survival, quality of life, anxiety, real-world data, personalized medicine

## Abstract

**Background**: Active surveillance (AS) represents a cornerstone of personalized medicine in uro-oncology, offering an individualized management strategy for low- and selected favourable intermediate-risk prostate cancer that aligns treatment intensity with patient-specific risk profiles; however, real-world adherence data from southern European academic centres remain scarce. This study aimed to evaluate AS discontinuation rates, reasons for transition to active treatment, and quality of life (QoL) in a single-centre Greek university hospital cohort. **Methods**: This is an observational retropective cohort study of patients enrolled in an AS protocol at the First Department of Urology, Aristotle University of Thessaloniki, between October 2016 and July 2022. Treatment-free survival (TFS) was estimated using Kaplan–Meier analysis. Associations between AS discontinuation and age at diagnosis, PSA level, and Charlson Comorbidity Index (CCI) were explored using univariable and multivariable Cox proportional hazards regression. QoL, erectile function, and anxiety were assessed cross-sectionally in patients remaining on AS using SF-12, IIEF-6, STAI-6, and MAX-PC. **Results**: Thirty-six patients were included (32 low-risk; 4 favourable intermediate-risk), with a median age of 69.5 years, median PSA of 6.92 ng/mL, and median CCI of 3. After a median follow-up of 24 months (IQR 21–45), 19 patients (52.8%) transitioned to active treatment; the median time to treatment was 21 months (IQR 17–34). Among the 10 patients with a known reason for discontinuation, 6 (31.6% of all discontinued) showed histopathological or clinical disease progression, 3 (15.8%) had a PSA increase alone, and 1 (5.3%) transitioned due to urinary symptoms; the reason was unknown in 9 cases (47.4%). In exploratory Cox regression, PSA ≥ 7.0 ng/mL was the only factor with complete documented output significantly associated with transition to active treatment (univariable HR 3.70, 95% CI 1.35–10.1, *p* = 0.011; multivariable HR 3.93, 95% CI 1.42–10.9, *p* = 0.008). Cross-sectional QoL assessment in 10 patients remaining on AS demonstrated median scores above established population norms for SF-12 and below clinical anxiety thresholds on STAI-6 and MAX-PC. **Conclusions**: In this single-centre cohort, AS discontinuation occurred early and at a rate higher than that of established international programmes, consistent with the institution’s initial AS experience. Higher PSA at diagnosis was the only factor with complete analytical documentation to be significantly associated with earlier transition.

## 1. Introduction

Prostate cancer is a biologically heterogeneous disease, ranging from clinically indolent to rapidly progressive, with most cases in the PSA era presenting as localized disease [1]. Opportunistic PSA screening, while enabling early diagnosis, carries a well-recognized risk of overdiagnosis and overtreatment, estimated to affect more than 50% of screen-detected cases [2]. For patients with low-risk and selected favourable intermediate-risk localized prostate cancer, active surveillance (AS) has emerged as the preferred management strategy across major international guidelines, including those of the European Association of Urology (EAU), the American Urological Association/American Society for Radiation Oncology (AUA/ASTRO), and the National Comprehensive Cancer Network (NCCN) [1,3,4,5,6].

AS aims to defer or avoid radical treatment and its associated morbidity, without compromising long-term cancer-specific survival. Long-term prospective AS cohorts have consistently demonstrated prostate cancer-specific mortality below 1% at 10 years, establishing AS as an oncologically safe management strategy in appropriately selected patients [7,8]. The core components of AS include serial PSA measurements, digital rectal examination, multiparametric MRI (mpMRI), and protocol-driven repeat prostate biopsies, although standardized protocols vary across institutions [9].

Despite growing global adoption, population-level data on real-world AS adherence, discontinuation rates, and reasons for transition to active treatment remain limited, particularly from southern and southeastern European settings. In a large population-based Canadian cohort (n = 8544), Timilshina et al. reported a 58% cumulative AS discontinuation rate at five years, with rates varying substantially between academic centres (24–41%) and community practices [10]. The Movember GAP3 consortium reported 43.6% discontinuation at five years across 21 centres in 12 countries [11], while PRIAS—a multinational AS registry including more than 150 centres—reported 52% discontinuation at five years [12].

Alongside oncological outcomes, the psychological impact of living with an untreated malignancy has received increasing attention. Patients on AS may experience anxiety related to disease progression, which can negatively affect QoL and may independently predict transition to active treatment in some series [13,14,15].

The present study reports one of the first systematic descriptions of real-world AS adherence and QoL outcomes from a Greek university centre. The aims were to (i) evaluate the rate of and reasons for AS discontinuation, and (ii) assess QoL, erectile function, and anxiety in patients remaining on AS.

## 2. Materials and Methods

### 2.1. Study Design and Setting

This is an observational retrospective cohort study conducted at the First Department of Urology, Aristotle University of Thessaloniki, with patients followed at the outpatient clinics of “G.Gennimatas” General Hospital of Thessaloniki, Greece. According to Greek legislation (Law 3418/2005 and subsequent amendments), non-interventional retrospective observational studies using anonymized or pseudonymized data collected during routine clinical care do not require formal Institutional Review Board approval or individual patient consent. Nevertheless, the study protocol was submitted to the Institutional Review Board of G.Gennimatas General Hospital for administrative notification and ethical assessment (reference number: 23; date: 12 February 2022), which confirmed that no further ethical approval was required under the applicable regulatory framework. All data were handled in accordance with the General Data Protection Regulation (EU 2016/679) and institutional privacy policies.

### 2.2. Eligibility Criteria

Patients diagnosed with localized prostate cancer and enrolled in the AS protocol between October 2016 and July 2022 were eligible for inclusion. Patients who declined informed consent or lacked adequate clinical records were excluded. The AS protocol applied inclusion criteria based on the AUA/ASTRO risk stratification system [6], as follows:

**Low-risk prostate cancer:**

- Clinical stage T1–T2a, ISUP Grade 1, PSA < 10 ng/mL, and life expectancy > 10 years.

**Favourable intermediate-risk prostate cancer:**

- ISUP Grade 1 and PSA 10–20 ng/mL, or clinical stage T2b–T2c with <50% positive biopsy cores; OR ISUP Grade 2 and PSA < 10 ng/mL and clinical stage T1–T2a and <50% positive biopsy cores; AND life expectancy > 10 years.

### 2.3. AS Monitoring Protocol and Data Collection

Patients were followed from the date of enrolment until the first occurrence of: (i) initiation of active treatment, (ii) death, or (iii) loss to follow-up. The AS monitoring protocol included regular PSA testing, digital rectal examination, mpMRI, and repeat prostate biopsies performed according to clinical indication and protocol specifications. Active treatment was defined as radical prostatectomy (open, laparoscopic, or robot-assisted), external beam radiotherapy (with or without androgen deprivation therapy [ADT]), or ADT alone.

For patients who transitioned to active treatment or were lost to follow-up, data on discontinuation and reasons for transition were collected via in-person interview at the last outpatient visit, telephone communication, electronic prescribing systems, or hospital medical records. Reasons for transition were categorized as: (1) histopathological or clinical disease progression; (2) PSA increase alone; (3) patient or physician decision in the absence of documented progression; (4) urinary symptoms; or (5) unknown.

### 2.4. Outcomes

The primary outcome was treatment-free survival (TFS), defined as the time from AS enrolment to initiation of active treatment, censored at death or last follow-up. Secondary outcomes were: (i) reasons for AS discontinuation; (ii) type of active treatment administered; and (iii) QoL, erectile function, and anxiety in patients remaining on AS.

### 2.5. Quality-of-Life Instruments

QoL was assessed cross-sectionally between October and December 2025, via structured telephone interviews, in patients who remained on AS at the time of contact and were clinically able to participate. Given the cross-sectional design and the absence of pre-AS baseline measurements, QoL findings are descriptive only. The following validated instruments were administered in their Greek-validated versions [16,17,18,19,20,21,22]:

- SF-12 (12-Item Short-Form Health Survey): generates a Physical Component Score (PCS) and Mental Component Score (MCS), each ranging from 0 to 100 (higher = better health). The Greek population mean is 50 (SD 10) for both components [16,17].
- IIEF-6 (International Index of Erectile Function, 6-item erectile function domain): range, 5–30; score ≤ 10 indicates severe erectile dysfunction [18,19].
- STAI-6 (State–Trait Anxiety Inventory, 6-item version): raw scores converted to STAI-20 equivalent by multiplying by 20/6 (range 20–80; ≥44 = high anxiety) [20,21].
- MAX-PC (Memorial Anxiety Scale for Prostate Cancer): range, 0–54; >27 = high anxiety; three subscales [22].

### 2.6. Statistical Analysis

TFS was estimated using the Kaplan–Meier method. Kaplan–Meier estimates are reported at 12 and 24 months as point estimates; estimates at 36 and 48 months are based on fewer than 10 patients at risk and must be interpreted with considerable caution. Because only one patient (2.8%) died from an unrelated cause during follow-up, the Kaplan–Meier method was used as the primary analysis.

Cox proportional hazards regression was performed to explore associations between AS discontinuation and age at diagnosis, PSA level, and CCI. Continuous predictors were categorized a priori as: age (<70 vs. ≥70 years), PSA (<7.0 vs. ≥7.0 ng/mL), and CCI (<3 vs. ≥3).

The PSA threshold of 7.0 ng/mL was selected a priori based on clinical plausibility rather than guideline alignment. In our centre’s early AS experience, this threshold represented the approximate median PSA of the cohort (6.92 ng/mL) and was used in prior internal audits to identify patients at higher risk of rapid progression. We acknowledge that this cutoff differs from the <10 ng/mL threshold used in AUA/ASTRO and EAU risk-stratification criteria, as well as from the 4–10 ng/mL range commonly applied in active surveillance protocols. The 7.0 ng/mL threshold was chosen to test whether a more restrictive PSA cutoff within the low-risk range would predict earlier transition in our specific population, recognizing that this is an exploratory analysis and that the findings require validation in larger cohorts using guideline-concordant thresholds.

The proportional hazards assumption was assessed using Schoenfeld residuals. With 19 events and three predictors, the events-per-variable (EPV) ratio was 6.3, below the recommended minimum of 10; all Cox results are accordingly exploratory and hypothesis-generating only. Univariable analyses were followed by a multivariable model including all three predictors simultaneously. QoL outcomes are summarized as medians and IQRs. All analyses were performed using R (version 4.3.0). Statistical significance was set at α = 0.05 (two-sided). The sample size was determined by the enrolment period (consecutive sampling).

## 3. Results

### 3.1. Cohort Characteristics

**Figure 1:**
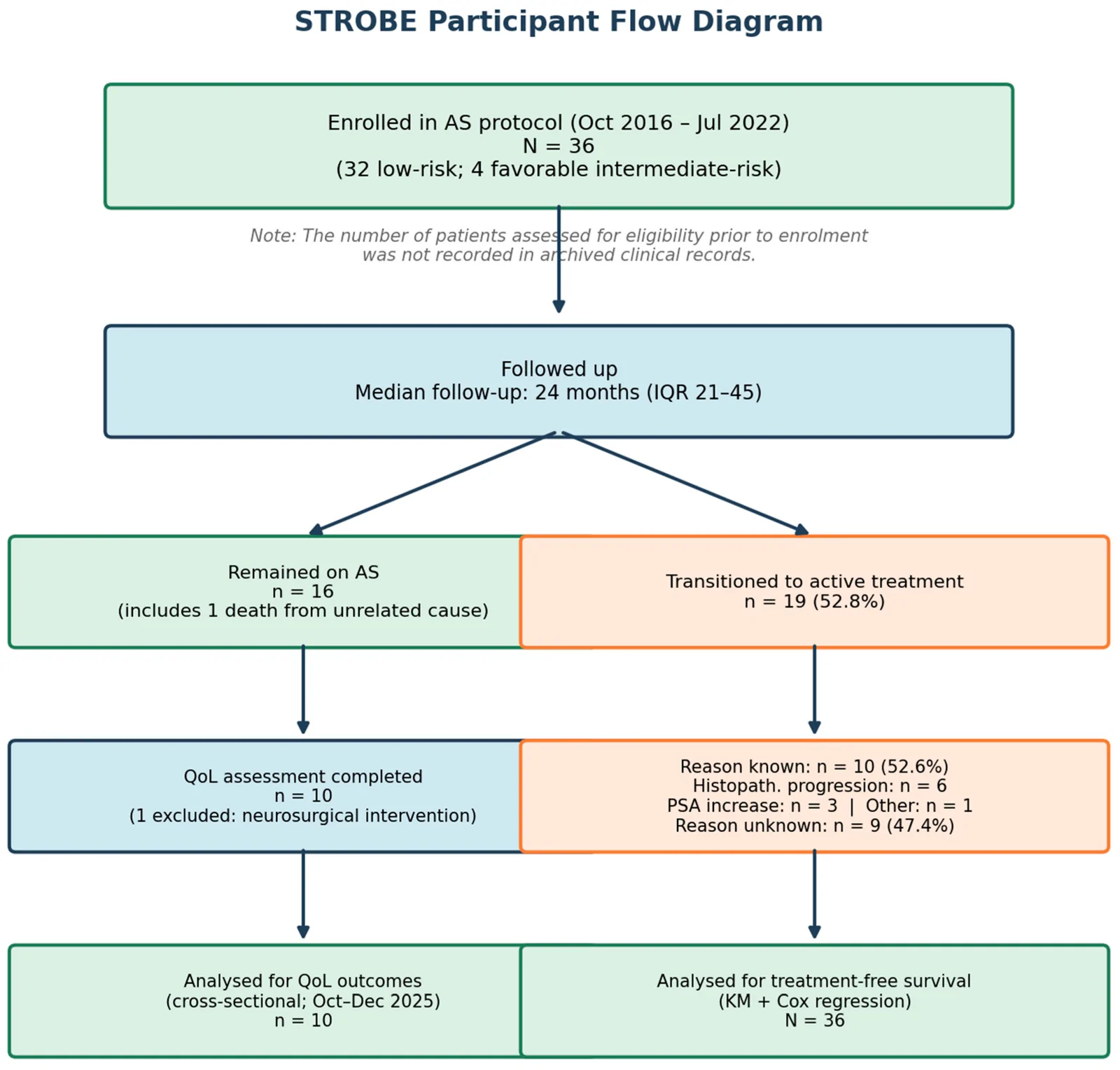
STROBE participant flow diagram. The number of patients assessed for eligibility prior to enrolment was not recorded in archived clinical records and is therefore not shown. AS = active surveillance; IQR = interquartile range; KM = Kaplan–Meier; QoL = quality of life.

A total of 36 patients with low-risk (n = 32, 88.9%) or favourable intermediate-risk (n = 4, 11.1%) localized prostate cancer were enrolled during the study period (Figure 1). Demographic and disease characteristics are summarized in Table 1. The median age at enrolment was 69.5 years, the median PSA at diagnosis was 6.92 ng/mL, and the median CCI was 3; individual-variable IQRs for these parameters were not consistently documented in primary clinical records. This reflects the retrospective nature of data extraction from an electronic health record system that lacked mandatory structured fields for these variables during the study period (2016–2022). Detailed histopathological baseline data (ISUP grade and clinical T stage distributions) were not available from archived records. One patient (2.8%) died during follow-up from a cause unrelated to prostate cancer. The median follow-up duration was 24 months (IQR 21–45 months).

### 3.2. Treatment-Free Survival and AS Discontinuation

**Figure 2:**
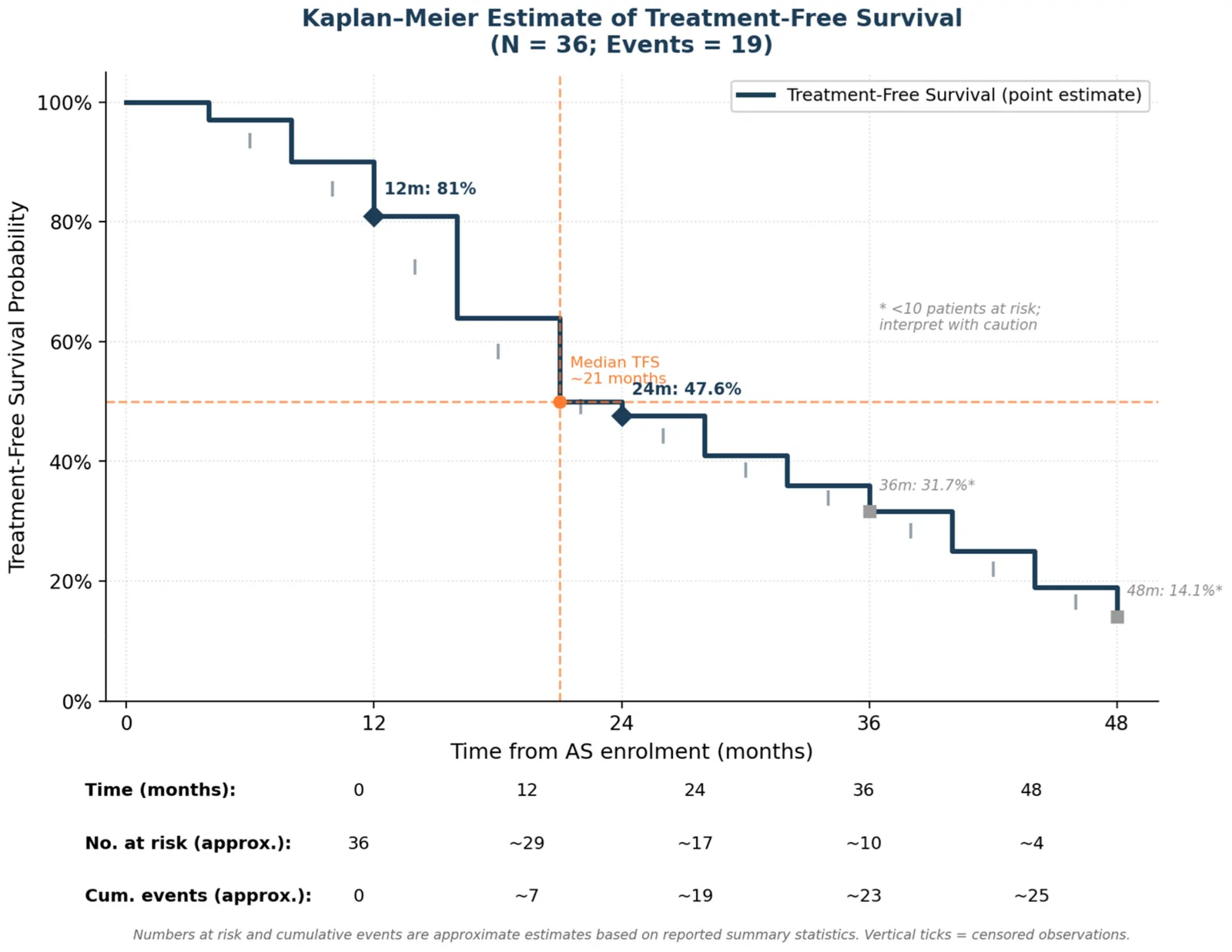
Kaplan–Meier estimate of treatment-free survival (n = 36; events = 19). Reported point estimates: 81% at 12 months and 47.6% at 24 months. Estimates at 36 and 48 months are based on 10 and 4 patients at risk, respectively, and should be interpreted with caution. Numbers at risk: 36 at 0 months; 29 at 12 months; 17 at 24 months; 10 at 36 months; 4 at 48 months. TFS = treatment-free survival.

Nineteen patients (52.8%) transitioned to active treatment during follow-up (Figure 1). The Kaplan–Meier TFS curve is presented in Figure 2. Estimated TFS was 81% (95% CI: 68–94%) at 12 months and 47.6% (95% CI: 31–64%) at 24 months. Estimates at 36 months (31.7%; 95% CI: 14–49%) and 48 months (14.1%; 95% CI: 0–32%) are based on 10 and 4 patients at risk, respectively, and must be interpreted with considerable caution. The median time to initiation of active treatment was 21 months (IQR 17–34 months).

Reasons for AS discontinuation and active treatments administered are presented in Table 2. Among the 19 patients who transitioned, the reason was known in 10 (52.6%): six (31.6% of all discontinued) had documented histopathological or clinical disease progression; three (15.8%) transitioned due to PSA increase alone; and one (5.3%) owing to urinary symptoms. The reason could not be determined in nine patients (47.4%), primarily due to incomplete clinical records. This substantial proportion of missing data is a major limitation: if transition in these patients was driven by unrecorded disease progression, our TFS estimates may be artificially prolonged; conversely, if transitions were driven by patient or physician preference in the absence of progression, the observed TFS would overestimate true progression-related discontinuation. Consequently, the primary outcome of treatment-free survival should be interpreted as a composite of oncological progression, clinical decision-making, and potentially unmeasured psychosocial factors, rather than as a pure measure of oncological failure. None of the patients who transitioned reported anxiety or psychological distress as the primary reason for discontinuation.

The majority received radical prostatectomy (n = 12, 63.2%), equally distributed among open, laparoscopic, and robot-assisted approaches (four each). Three patients (15.8%) received radiotherapy combined with ADT, one (5.3%) radiotherapy alone, and three (15.8%) ADT alone (see Table 2 footnote).

### 3.3. Predictors of Transition to Active Treatment

The results of the exploratory Cox proportional hazards regression for PSA are presented in Table 3. The proportional hazards assumption was assessed using Schoenfeld residuals. In the original analysis, age ≥ 70 years and CCI ≥ 3 were reported as not significantly associated with transition to active treatment in either analysis; however, as the numerical outputs for these variables could not be retrieved from archived records, they are not formally tabulated here.

In univariable analysis, patients with PSA ≥ 7.0 ng/mL had a significantly higher risk of transitioning compared with patients with PSA < 7.0 ng/mL (HR 3.70; 95% CI 1.35–10.1; *p* = 0.011). This association remained significant in multivariable analysis (HR 3.93; 95% CI 1.42–10.9; *p* = 0.008). Given an EPV ratio of 6.3, these results are exploratory; the wide confidence intervals reflect statistical uncertainty inherent to this sample size.

### 3.4. Quality-of-Life Outcomes

Ten patients who remained on AS participated in the QoL assessment; one was excluded owing to recent neurosurgical intervention and poor clinical status. QoL results are presented in Table 4. These findings reflect a cross-sectional assessment at a single, non-predefined timepoint in a survivor cohort and should not be interpreted as evidence of a longitudinal effect of AS on QoL.

Median SF-12 scores were 56.41 (PCS; IQR 55.26–57.70) and 56.42 (MCS; IQR 54.83–60.70), both above the Greek population mean of 50. The median IIEF-6 score was 21.5 (IQR 12.0–25.5); three patients (30%) scored ≤ 10, indicating severe erectile dysfunction. The median STAI-6 score (STAI-20 equivalent) was 26.67 (IQR 20.83–35.00); one patient (10%) met the high-anxiety threshold of ≥44. The median MAX-PC score was 9.50 (IQR 2.75–17.75); no patient exceeded the clinical threshold of >27.

## 4. Discussion

This single-centre cohort study is among the first to report real-world AS adherence and QoL data from southeastern Europe. Approximately 53% of enrolled patients transitioned to active treatment after a median follow-up of 24 months, a rate consistent with early-experience settings but exceeding that of established international AS programmes.

In the large population-based Canadian cohort reported by Timilshina et al. (n = 8544), cumulative AS discontinuation reached 58% at five years, with rates varying markedly by setting: 24–41% in academic centres versus higher rates in community practices [10]. Our finding of 52.8% discontinuation at a median of 24 months likely reflects the institution’s initial AS experience—meaningful recruitment began only after 2018—alongside less strict inclusion criteria and evolving counselling practices. The PRIAS study reported 52% discontinuation at five years across more than 150 heterogeneous international centres [12], the GAP3 consortium reported 43.6% at five years [11], and the Swedish national cohort demonstrated 36% over the same period [7]. The Johns Hopkins AS programme, applying particularly strict inclusion criteria, reported 81% TFS at two years and 59% at five years [7]. Together, these comparisons confirm that AS discontinuation rates are strongly influenced by institutional experience, inclusion criteria stringency, and follow-up intensity.

The high rate of unknown discontinuation reasons (9/19 patients; 47.4%) is both a significant data limitation and a clinically informative finding: it demonstrates that without structured, prospective data capture integrated into the AS workflow, retrospective retrieval of transition reasons is unreliable. Future AS programmes should implement mandatory electronic recording of all protocol milestones and transition events at the time of occurrence.

In exploratory Cox regression, higher PSA at diagnosis (≥7.0 vs. <7.0 ng/mL) was the only factor with complete documented analytical output significantly associated with transition to active treatment. Age and CCI were reported as non-significant in the original analysis; as their numerical outputs could not be verified from archived records, no formal inference can be drawn regarding these variables beyond noting their reported non-significance. Despite these limitations, the PSA association is consistent with multiple larger cohorts: Timilshina et al. [10], Loeb et al. [7], and Cooley et al. in the SPORE cohort (n = 7279) [23] all identified higher baseline PSA as a significant predictor of earlier transition.

Over the past decade, increasing evidence has established that AS does not adversely affect QoL in the short or medium term [13,24]. Alberti et al. found comparable or better patient-reported outcomes after AS versus radical treatments across prospective comparative studies [13], and Thompson et al. (2023) reported QoL comparable to non-cancer populations over ≥5 years [24]. The Finnish PRIAS cohort demonstrated no deterioration in QoL over up to 11 years of follow-up [25]. In the START cohort study, patients who underwent radical prostatectomy reported significantly worse sexual function and urinary continence than AS patients at 36 months [26]. The preservation of QoL in patients remaining on AS reinforces the personalized medicine principle of aligning management intensity with patient priorities, particularly for those who place high value on maintaining sexual and urinary function [24,25,26].

In the present cohort, cross-sectional QoL assessment in 10 patients remaining on AS demonstrated median SF-12 scores above the Greek population norm and anxiety scores below clinical thresholds. These descriptive findings are broadly consistent with the established literature but cannot support longitudinal or causal conclusions, given the absence of baseline measurements, the non-predefined assessment timepoint, and the survivor selection inherent to assessing only those who remained on AS.

### Limitations

The following limitations must be considered. First, the small sample size (n = 36) and low event count (n = 19) render all statistical analyses exploratory, with an EPV ratio of 6.3 precluding reliable multivariable inference. Second, the study was conducted at a single centre, limiting external validity. Third, the reason for AS discontinuation was unknown in 47.4% of transitioned patients, representing substantial missing data that may have introduced differential misclassification. Because unrecorded progression events could not be distinguished from patient-initiated or physician-initiated transitions, the primary outcome (treatment-free survival) likely reflects a mixture of oncological and non-oncological drivers. This ambiguity limits causal inference regarding predictors of progression and may bias hazard ratio estimates if the missingness mechanism is related to the predictors under study. Fourth, detailed histopathological baseline data (ISUP grade and clinical T stage) were not retrievable from archived records. Fifth, the cohort included four favourable intermediate-risk patients alongside 32 low-risk patients; their small number precluded subgroup analysis. Sixth, the median follow-up of 24 months limits long-term conclusions. Seventh, QoL was assessed cross-sectionally in 10 survivor patients without baseline measurements, introducing survivor selection bias. Eighth, hazard ratios for age and CCI could not be retrieved from archived analytical records. Ninth, the 7.0 ng/mL PSA categorization is not aligned with major guideline thresholds (e.g., <10 ng/mL in AUA/ASTRO and EAU criteria) and was applied a priori based on clinical plausibility; this limits comparability with international cohorts and may reduce generalizability. Finally, PSA kinetics (doubling time, velocity), PSA density, and mpMRI findings were not available as analytical predictors.

This cohort study describes real-world AS adherence outcomes from a Greek university centre within a personalized medicine framework, representing one of the first such reports from south-eastern Europe. Prospective multi-centre studies with standardized data capture, pre-specified QoL assessment intervals with baseline measurements, and sample sizes sufficient for adequately powered multivariable analysis are needed to generate robust, personalized evidence for optimizing AS implementation in Greece and comparable settings.

## 5. Conclusions

This cohort study describes real-world AS adherence outcomes from a Greek university centre, representing one of the first such reports from south-eastern Europe and contributing to the personalized medicine evidence base by evaluating individualized risk factors and patient-reported outcomes. These exploratory findings underscore the importance of careful patient selection, structured multidisciplinary counselling, systematic prospective data collection, and adequate institutional experience in establishing AS programmes. By demonstrating that higher baseline PSA predicts earlier transition and that QoL remains satisfactory in continuing patients, this study supports the personalisation of AS protocols to individual risk profiles and patient priorities.

## Figures and Tables

**Table 1:** Baseline patient and disease characteristics.

Characteristic	All Patients (n = 36)
Age at diagnosis, years, median	69.5
PSA at diagnosis, ng/mL, median	6.92
Charlson Comorbidity Index, median	3
Risk group, n (%)	
Low-risk	32 (88.9)
Favourable intermediate-risk	4 (11.1)
ISUP Grade and Clinical T Stage, n (%)	Not available from archived records
Follow-up duration, months, median (IQR)	24 (21–45)
Transitioned to active treatment, n (%)	19 (52.8)
Time to active treatment, months, median (IQR)	21 (17–34)
Death from unrelated cause, n (%)	1 (2.8)

Detailed distributions of ISUP grade and clinical T stage by individual patients were not retrievable from archived clinical records at the time of analysis. Individual-variable interquartile ranges for age, PSA, and CCI were not consistently documented in the primary clinical records retrieved for this retrospective analysis. Detailed distributions of ISUP grade and clinical T stage by individual patients were similarly unavailable because the electronic health record system in use during the enrolment period (2016–2022) did not mandate structured capture of these variables in the outpatient urology module; data were recorded in free-text discharge summaries that were not fully digitized for all patients at the time of study conduct. IQR = interquartile range; ISUP = International Society of Urological Pathology; PSA = prostate-specific antigen.

**Table 2:** Reasons for AS discontinuation and active treatmentadministered.

Part A: Reasons for AS Discontinuation			
Reason	n	% of All Discontinued (n = 19)	% of Known Reasons (n = 10)
Histopathological or clinical disease progression	6	31.6	60.0
PSA increase alone	3	15.8	30.0
Other (urinary symptoms)	1	5.3	10.0
Unknown	9	47.4	—
Total	19	100.0	—
**Part B: Active Treatment Administered**			
**Active Treatment**	**n**	**% (n = 19)**	
Radical prostatectomy (total)	12	63.2	
Open radical prostatectomy	4	21.1	
Laparoscopic radical prostatectomy	4	21.1	
Robot-assisted radical prostatectomy	4	21.1	
Radiotherapy + androgen deprivation therapy	3	15.8	
Radiotherapy alone	1	5.3	
Androgen deprivation therapy alone a	3	15.8	
Total	19	100.0	

ADT alone was administered in three patients owing to advanced age, significant comorbidity burden, and patient preference following multidisciplinary discussion; curative-intent treatment was deemed clinically feasible. ADT = androgen deprivation therapy; PSA = prostate-specific antigen.

**Table 3:** Exploratory Cox proportional hazards regression—PSA as predictor of transition to active treatment.

Variable	Univariable HR (95% CI)	*p*-Value	Multivariable HR (95% CI)	*p*-Value
PSA ≥ 7.0 vs. <7.0 ng/mL	3.70 (1.35–10.1)	0.011	3.93 (1.42–10.9)	0.008

Results are from exploratory Cox proportional hazards regression (EPV = 6.3; findings are hypothesis-generating only). Hazard ratios for age and CCI were not retrievable from archived analytical records and are not reported; both were reported as non-significant in the original analysis. CI = confidence interval; EPV = events per variable; HR = hazard ratio; PSA = prostate-specific antigen.

**Table 4:** Cross-sectional quality-of-life and anxiety assessment results (n = 10).

Instrument	Domain	Median	IQR	Clinical Threshold	Patients at or Above Threshold, n (%)
SF-12	Physical Component Score (PCS)	56.41	55.26–57.70	<50 (below population norm)	1 (10.0)
SF-12	Mental Component Score (MCS)	56.42	54.83–60.70	<50 (below population norm)	2 (20.0)
IIEF-6	Erectile Function	21.5	12.00–25.50	≤10 (severe ED)	3 (30.0)
STAI-6 a	General Anxiety	26.67	20.83–35.00	≥44 (high anxiety)	1 (10.0)
MAX-PC	Cancer-Related Anxiety (total)	9.50	2.75–17.75	>27 (high anxiety)	0 (0.0)

STAI-6 scores converted to STAI-20 equivalent (raw score × 20/6; range 20–80). Greek population norm for SF-12: mean 50, SD 10. ED = erectile dysfunction; IIEF-6 = 6-item erectile function domain; IQR = interquartile range; MAX-PC = Memorial Anxiety Scale for Prostate Cancer; SF-12 = 12-Item Short-Form Health Survey; STAI-6 = State–Trait Anxiety Inventory (6-item version).

## Data Availability

The data presented in this study are available on reasonable request from the corresponding author. The data are not publicly available, due to privacy restrictions.

## Abbreviations

The following abbreviations are used in this manuscript:

ADT	Androgen Deprivation Therapy
AS	Active Surveillance
AUA	American Urological Association
ASTRO	American Society for Radiation Oncology
CCI	Charlson Comorbidity Index
CI	Confidence Interval
EAU	European Association of Urology
EPV	Events Per Variable
HR	Hazard Ratio
IIEF-6	International Index of Erectile Function—6-item version
IQR	Interquartile Range
ISUP	International Society of Urological Pathology
MAX-PC	Memorial Anxiety Scale for Prostate Cancer
mpMRI	Multiparametric Magnetic Resonance Imaging
MCS	Mental Component Score
NCCN	National Comprehensive Cancer Network
PCS	Physical Component Score
PSA	Prostate-Specific Antigen
QoL	Quality of Life
SF-12	12-Item Short-Form Health Survey
STAI-6	State–Trait Anxiety Inventory—6-item version
TFS	Treatment-Free Survival
